# Correlation of the Size of Adenoids With Impedance Audiometry Findings

**DOI:** 10.7759/cureus.62453

**Published:** 2024-06-15

**Authors:** Anvitha Suresh, Gundappa Mahajan, James Thomas, Manu Babu

**Affiliations:** 1 Otolaryngology - Head and Neck Surgery, Dr. D. Y. Patil Medical College, Hospital and Research Centre, Dr. D. Y. Patil Vidyapeeth, Pune, IND; 2 Otorhinolaryngology - Head and Neck Surgery, Dr. D. Y. Patil Medical College, Hospital and Research Centre, Dr. D. Y. Patil Vidyapeeth, Pune, IND

**Keywords:** tympanometry, impedance audiometry, serous otitis media, otitis media with effusion, adenoid hypertrophy

## Abstract

Introduction

Adenoid tissue is part of the first line of immunity of the upper aero-digestive tract. It is located in the postero-superior wall of the nasopharynx behind the choana. Adenoid hypertrophy, a common childhood disorder, significantly contributes to the pathogenesis of otitis media with effusion (OME), which is the leading cause of hearing impairment in young children. This condition can result in delayed speech, poor academic performance, and language development issues. Assessing the size of the adenoids and their correlation with OME is crucial, as undiagnosed cases can lead to complications such as atelectasis of the tympanic membrane and cholesteatoma. Clinical examination of the nose alone is often insufficient, and children do not cooperate for nasal endoscopy. Therefore, a lateral radiograph of the skull is considered the most reliable method for assessing the adenoid size. The size of the adenoids can affect Eustachian tube patency, which is reflected in the results of impedance audiometry. This study aimed to correlate the size of adenoids with impedance audiometry findings.

Methods

This cross-sectional observational study was conducted in the Department of Otorhinolaryngology of a tertiary care hospital from October 1, 2022, to March 31, 2024. A sample size of 50 patients was taken for the study. The inclusion criterion of selection of the patients included patients aged 3 to 15 years, who suffered from recurrent attacks of upper respiratory tract infections, particularly those with adenoid facies confirmed by X-ray with a non-perforated tympanic membrane. Exclusion criteria encompassed patients below 3 or above 15 years, and those with acute or chronic suppurative otitis media, craniofacial anomalies, or nasal pathologies like polyps. Adenoids were graded using X-ray imaging of the nasopharynx, and correlations between the adenoid size and impedance audiometry findings, such as middle ear pressure and compliance, were analyzed.

Results

The study assessed the relationship between the adenoid size and impedance audiometry findings, focusing on middle ear pressure and compliance, as well as the occurrence of OME. The results indicated a significant decline in middle ear pressure with increasing adenoid grades. Specifically, adenoid grade 1 had an average pressure of -3.50 daPa, while grade 4 had the lowest average pressure at -119.72 daPa. This trend was statistically significant with a p-value of 0.00042. Similarly, compliance values also decreased with higher adenoid grades. Grade 1 had an average compliance of 0.64 ml, whereas grade 4 had the lowest average compliance at 0.28 ml. This relationship was statistically significant, as indicated by a p-value of 0.0048. Additionally, the analysis showed that a significant majority of patients with enlarged adenoids also presented with OME, highlighting a strong association between adenoid hypertrophy and this condition.

Conclusion

The study concluded that larger adenoids were associated with lower middle ear pressure and reduced compliance. Additionally, a significant majority of patients with enlarged adenoids also had OME. This underscores the importance of evaluating adenoid hypertrophy in the context of OME due to its potential impact on childhood hearing and development.

## Introduction

Adenoid tissue serves as a key component of the upper aero-digestive tract's initial immune defense. Wilhelm Meyer introduced the term "adenoid" to refer to what he called ‘nasopharyngeal vegetations’. The adenoid forms the uppermost part of the ring of submucosal lymphoid tissues in the pharynx (Waldeyer’s ring). It is located in the postero-superior wall of the nasopharynx behind the choana.

Adenoids grow rapidly during infancy and reach a plateau between the ages of 2 and 14. After the age of 15, they begin to regress [1]. On average, adenoids are the largest when a child is about 7 years old. The size of adenoids varies among children and within the same child as they grow. Notably, between the ages of 3 and 5, the nasopharyngeal soft tissue experiences significant growth, causing the airway to narrow. Following this period, the nasopharynx continues to grow while the soft tissue size remains relatively stable, resulting in an increase in the airway size.

Adenoid hypertrophy is a common disorder in children and significantly contributes to the development of otitis media with effusion, the leading cause of hearing impairment in young children. This condition can result in delayed speech and poor academic and language development. Otitis media with effusion (OME) is most frequently seen in children aged 4 to 8 years. If left undiagnosed, it can cause silent complications, such as damage to the tympanic membrane due to the local inflammatory response triggered by leukotrienes, prostaglandins, and arachidonic acid metabolites in the middle ear fluid, as noted by Yellon et al. [2]. Additionally, negative pressure in the middle ear can cause focal retraction pockets or atelectasis of the tympanic membrane, potentially leading to cholesteatoma. Therefore, it is important to evaluate adenoid hypertrophy for the presence of otitis media with effusion.

Clinical examination of children with nasal obstruction is often inconclusive for adenoids. Anterior rhinoscopy may be normal, with increased secretions or inferior turbinate hypertrophy. Examination of the nasopharynx with a posterior rhinoscopy mirror is difficult in children. Also, complete visualization of adenoids is not possible in diagnostic nasal endoscopy. Thus, in many children, it is difficult to assess the size of adenoids using these methods. The most reliable way of assessing the size of the adenoids is to take a lateral radiograph of the skull to visualize the nasopharynx. The adenoid shadow reveals the measure of the absolute size of the adenoids and its relation to the size of the airway. The extent of nasopharyngeal airway obstruction was evaluated using the adenoid-nasopharyngeal ratio, determined from a lateral view X-ray of the skull. The child was positioned erect with the head fixed in the Frankfort horizontal plane. The adenoid-nasopharyngeal ratio is categorized as grade I (0- 25%), grade II (25- 50%), grade III (50-75%), and grade IV (75-100%) [3].

Hypertrophy of the adenoids can impinge on the proximal opening of the Eustachian tube thus affecting its patency, which is reflected in the results of impedance audiometry.

## Materials and methods

This cross-sectional observational study was carried out in the Department of Otorhinolaryngology in a tertiary care hospital (Dr. D. Y. Patil Medical College, Hospital and Research Centre, Pune). The study period spanned from 01 October 2022 to 31 March 2024. The sample size of this study was 50.

The inclusion criterion of selection of the patients included patients aged 3 to 15 years, who suffered from recurrent attacks of upper respiratory tract infection particularly those with adenoid facies confirmed by X-ray with a non-perforated tympanic membrane. Patients were excluded if they were below 3 years or above 15 years of age, had acute or chronic suppurative otitis media, craniofacial anomalies, or nasal pathologies such as polyps and gross deviated nasal septum.

Initially, a detailed history was taken from patients presenting with mouth breathing, snoring, ear complaints, recurrent upper respiratory tract infections, and adenoid facies. Extra-oral features of adenoid facies included pinched nostrils, a short upper lip, an open mouth posture, an elongated face, a steep angle of the mandible, a vacant expression, and retrognathism. Intra-oral features included prominent upper teeth, a high-arched palate, crowded teeth, a narrow upper alveolus, and a hypoplastic maxilla.

**Figure 1:**
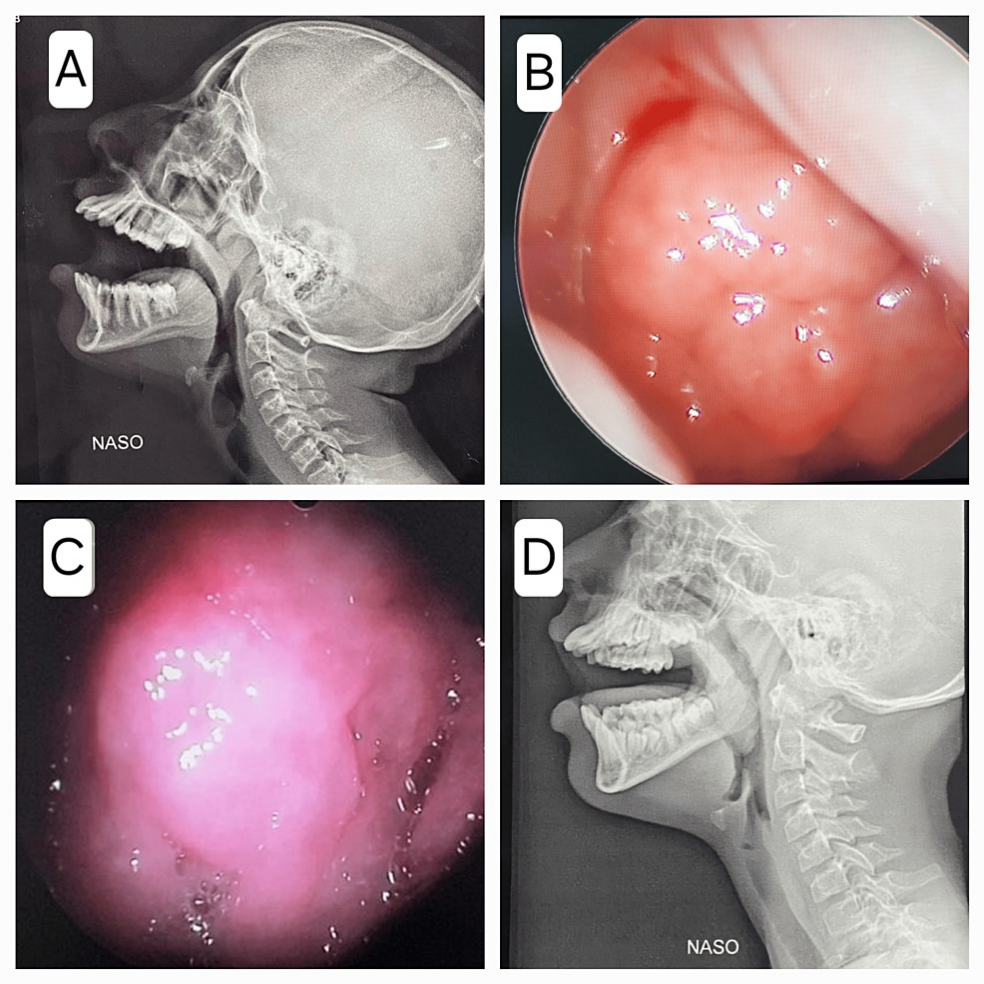
B shows the endoscopic image of the adenoid and A is its corresponding X-ray. C shows the endoscopic image of the adenoid and D is its corresponding X-ray.

Following this, a comprehensive examination of the ear, nose, throat, oral cavity, face, and neck, including an otoscopic examination, was carried out on each patient in the outpatient department of the hospital. Patients with adenoid hypertrophy on radiological examination were included in the study. The adenoids were graded based on their size as visualized in the X-ray of the skull (lateral view) (Figure 1), and the correlation between the adenoid size and impedance audiometry findings, such as middle ear pressure and compliance, was analyzed. The occurrence of OME with respect to the adenoid size was also studied. All details were recorded in a pre-prepared proforma.

Institute ethics committee approval was obtained before the start of the study. Participants were informed about the purpose and details of the study, and written informed consent was obtained prior to their enrolment.

## Results

The research encompassed individuals from Maharashtra, India, aged between 3 and 15 years, with a majority being in the 10 to 13 age bracket. Of the participants, 35.85% were female, while 64.15% were male. To assess the relation of the size of adenoids with middle ear pressure, the average middle ear pressure of the patients was initially found out by averaging the left and right ear values. Then a regression was performed in Excel to analyze the statistical significance of the variables.

**Figure 2:**
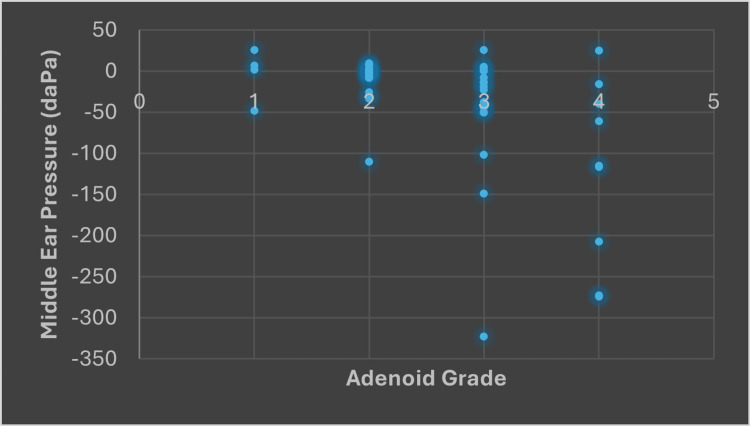
Relationship between the adenoid grade and middle ear pressure.

Table 1 illustrates the minimum, maximum, and average values of middle ear pressure for various adenoid grades. For adenoid grade 1, the middle ear pressure ranges from a minimum of -48.50 daPa (dekapascal) to a maximum of 25.50 daPa, with an average pressure of -3.50 daPa. In grade 2, the pressure values drop further, with a minimum of -110 daPa and a maximum of 9.50 daPa, resulting in an average of -10.87 daPa. Grade 3 presents an even more significant decline, with the minimum pressure at -322.50 daPa and the maximum still at 25.50 daPa, leading to an average of -46.81 daPa. Finally, grade 4 shows a minimum middle ear pressure of -274 daPa, a maximum of 25 daPa, and the lowest average pressure among all grades at -119.72 daPa. This data suggests a clear trend of worsening middle ear pressure as the adenoid size increases as shown in Figure 2.

**Table 1:** A range of values of middle ear pressure for various adenoid grades.

Adenoid grade	Minimum middle ear pressure (daPa)	Maximum middle ear pressure (daPa)	Average middle ear pressure (daPa)
1	-48.50	25.50	-3.50
2	-110	9.50	-10.87
3	-322.50	25.50	-46.81
4	-274	25	-119.72

The p-value for this experiment came out as 0.00042, which is a very small value. This indicates that there is statistical significance in the relationship between middle ear pressure and the size of adenoids.

To assess the relationship between the size of adenoids and compliance, the average compliance of the ears was found by averaging the left and right ear values. Then, like before, a regression was performed to analyze the statistical significance of the variables.

**Figure 3:**
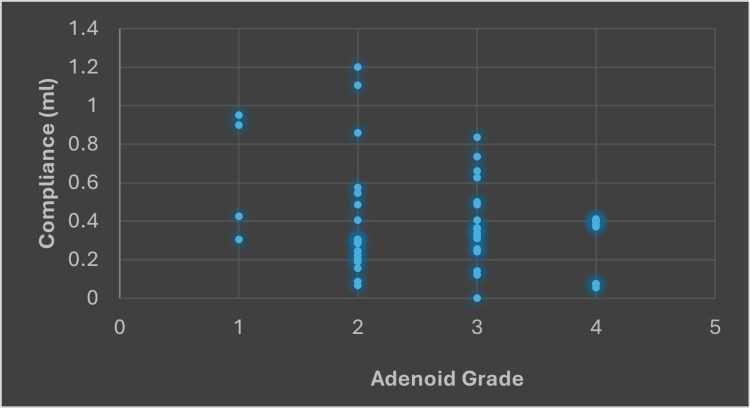
Relationship between the adenoid grade and compliance.

Figure 3 provides detailed data on the compliance values across different adenoid grades. For adenoid grade 1, the minimum compliance value is 0.30 ml, the maximum is 0.95 ml, and the average is 0.64 ml. In grade 2, compliance values range from a minimum of 0.06 ml to a maximum of 1.10 ml, with an average of 0.41 ml. Grade 3 shows a minimum compliance value of 0 ml, a maximum of 0.83 ml, and an average of 0.38 ml. Lastly, grade 4 has a minimum value of 0.05 ml, a maximum of 0.41 ml, and the lowest average compliance at 0.28 ml. As shown in Table 2, this data indicates that as the adenoid grade increases, there is a general trend toward lower average compliance values.

**Table 2:** A range of values of compliance for various adenoid grades.

Adenoid grade	Minimum compliance (ml)	Maximum compliance (ml)	Average compliance (ml)
1	0.30	0.95	0.64
2	0.06	1.10	0.41
3	0	0.83	0.38
4	0.05	0.41	0.28

In this study, the p-value came at 0.0048. This indicates that there is statistical significance in the relationship between compliance and the adenoid grade, as per the analyzed data.

**Figure 4:**
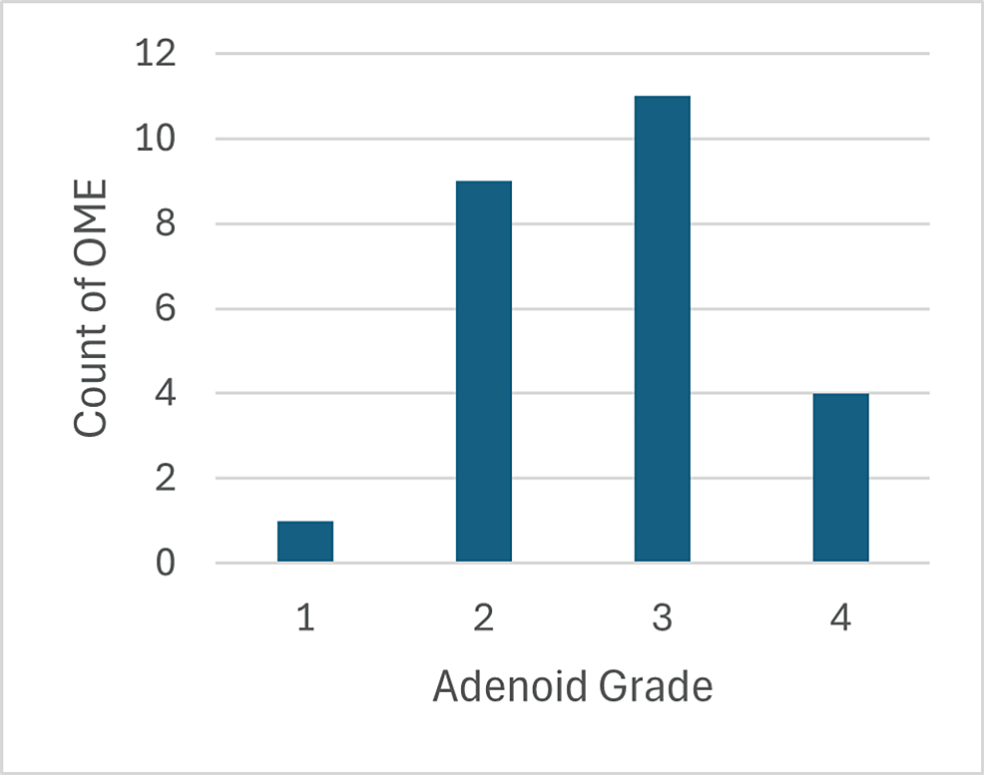
Count of otitis media with effusion cases among adenoid grades. OME: Otitis media with effusion

The study also indicated that a significant majority of the patients having adenoids showed the presence of OME as shown in Figure 4.

## Discussion

The study's findings hold clinical implications for the management of patients with adenoid hypertrophy and associated middle ear disorders. Understanding the relationship between adenoid size and middle ear pressure can aid clinicians in predicting the likelihood of OME development or recurrence in patients with adenoid hypertrophy. Patients with larger adenoids may warrant closer monitoring for OME and timely interventions such as adenoidectomy to alleviate middle ear pressure and improve outcomes.

Furthermore, the association between adenoid size and compliance suggests that other factors, such as Eustachian tube function and mucosal inflammation, have a more substantial role in middle ear compliance. Clinicians should consider a holistic approach to evaluating middle ear function, incorporating factors beyond adenoid size alone.

The study by Rajashekar et al. and Maw et al. primarily focused on postoperative changes in middle ear parameters following adenoidectomy in children with adenoid hypertrophy. While their emphasis was on assessing the efficacy of surgical intervention, their findings underscored the high prevalence of OME in patients with adenoid hypertrophy. This resonates with the current study's emphasis on the clinical significance of adenoid size in predisposing individuals to middle ear dysfunction, particularly OME [4,5].

In contrast, Bhat et al. investigated the association of asymptomatic OME in patients with adenoid hypertrophy. Their study highlighted the importance of impedance audiometry in diagnosing fluid in the middle ear, even in cases where symptoms may not be apparent. This aligns with the current study's focus on elucidating the relationship between adenoid size and middle ear parameters, emphasizing the need for comprehensive evaluation in all patients with adenoid hypertrophy [6].

Furthermore, Günel et al. explored the effect of adenoid hypertrophy on tympanometric findings in children without hearing loss. While their study did not directly assess surgical interventions, their findings indicated a significant decrease in negative middle ear pressure following adenoidectomy. This suggests a potential therapeutic role for adenoidectomy in alleviating middle ear dysfunction, complementing the current study's emphasis on the relationship between adenoid size and middle ear parameters [7].

On the other hand, in the study by Fujioka et al., radiographic evaluation of adenoid size in children provided valuable insights into adenoid growth patterns. While their study did not directly assess middle ear parameters, their findings shed light on anatomical considerations that may indirectly influence middle ear function. By incorporating radiographic assessments, their study complemented the current study's emphasis on understanding the anatomical basis of middle ear disorders [8].

Paradise et al. undertook an examination of the prevalence and risk factors of otitis media in infants, indirectly related to the occurrence of OME. However, it is worth noting that their study primarily focused on infants, whereas the present study targets children aged 3 to 15 years [9].

Durgut et al. and Tawab et al., on the other hand, explored the effect of adenoid hypertrophy on hearing thresholds and middle ear fluid viscosity in OME, respectively, similar to the current study's focus on impedance audiometry findings. Nevertheless, their studies may focus more specifically on hearing thresholds or middle ear fluid viscosity rather than direct correlations with adenoid size [10,11].

Overall, the current study adds to the existing literature by providing further evidence of the relationship between adenoid size and middle ear pressure. By elucidating this relationship, the study contributes to our understanding of the pathophysiology of middle ear disorders and highlights the potential role of adenoidectomy in managing these conditions. Further research incorporating larger sample sizes and longitudinal designs is warranted to validate these findings and inform clinical practice effectively.

Limitations of the study include its cross-sectional design, which limits the establishment of causality, and the relatively small sample size, which may impact the generalizability of the findings. The study only considered the anteroposterior extent of the adenoids as seen in the lateral radiograph of the skull and not the lateral extent of it. Additionally, the study focused solely on the relationship between adenoid size and middle ear parameters, without considering other potential confounding variables.

## Conclusions

This study aimed to assess the correlation between the size of adenoids and impedance audiometry findings, specifically focusing on middle ear pressure and compliance, as well as the occurrence of OME. For middle ear pressure, the data showed a clear trend of worsening pressure as the adenoid grade increases. This indicates that larger adenoids are associated with more negative middle ear pressure, which can lead to complications such as focal retraction pockets or atelectasis of the tympanic membrane. Similarly, compliance values decrease with higher adenoid grades. Furthermore, the analysis showed that a significant majority of patients with enlarged adenoids also presented with otitis media with effusion.

Overall, the study highlights the importance of evaluating adenoid size in children with ear complaints. Enlarged adenoids are significantly correlated with negative middle ear pressure and decreased compliance, both of which contribute to the development of otitis media with effusion. This emphasizes the need for careful clinical assessment and potential intervention in pediatric patients with adenoid hypertrophy to prevent associated complications and promote better auditory and developmental outcomes. Further research may explore other contributing factors to middle ear pressure variability and the broader clinical implications of adenoid hypertrophy.
